# Forming new sex partnerships while overseas: findings from the third British National Survey of Sexual Attitudes & Lifestyles (Natsal-3)

**DOI:** 10.1136/sextrans-2015-052459

**Published:** 2016-06-06

**Authors:** Clare Tanton, Anne M Johnson, Wendy Macdowall, Jessica Datta, Soazig Clifton, Nigel Field, Kirstin R Mitchell, Kaye Wellings, Pam Sonnenberg, Catherine H Mercer

**Affiliations:** 1 Research Department of Infection & Population Health, University College London, London, UK; 2 Department of Social & Environmental Health Research, London School of Hygiene and Tropical Medicine, London, UK; 3 NatCen Social Research, London, UK; 4 MRC/CSO Social & Public Health Sciences Unit, University of Glasgow, Glasgow, UK

**Keywords:** EPIDEMIOLOGY (GENERAL), SEXUAL BEHAVIOUR, TRAVEL

## Abstract

**Objectives:**

Travelling away from home presents opportunities for new sexual partnerships, which may be associated with sexually transmitted infection (STI) risk. We examined the prevalence of, and factors associated with, reporting new sexual partner(s) while overseas, and whether this differed by partners’ region of residence.

**Methods:**

We analysed data from 12 530 men and women aged 16–74 years reporting ≥1 sexual partner(s) in the past 5 years in Britain's third National Survey of Sexual Attitudes and Lifestyles (Natsal-3), a probability survey undertaken 2010–2012.

**Results:**

9.2% (95% CI 8.3% to 10.1%) of men and 5.3% (4.8% to 5.8%) of women reported new sexual partner(s) while overseas in the past 5 years. This was strongly associated with higher partner numbers and other sexual and health risk behaviours. Among those with new partners while overseas, 72% of men and 58% of women reported partner(s) who were not UK residents. Compared with those having only UK partners while abroad, these people were more likely to identify as ‘White Other’ or ‘Non-White’ (vs White British ethnicity), report higher partner numbers, new partners from outside the UK while in the UK and paying for sex (men only) all in the past 5 years. There was no difference in reporting STI diagnosis/es during this time period.

**Conclusions:**

Reporting new partners while overseas was associated with a range of sexual risk behaviours. Advice on sexual health should be included as part of holistic health advice for all travellers, regardless of age, destination or reason for travel.

## Introduction

Over the past two decades, international travel has increased considerably with people travelling more frequently and to a wider range of destinations. In 2014, an estimated 60.1 million trips abroad were made by UK residents.^1^ People, including migrants to the UK visiting their country of origin, travel for many reasons—leisure, business, study, to visit family and friends.^1–3^ Some also travel expressly to have sex.^4^ ^5^ Even if sex is not an explicit motivation for travel, sexual encounters may be facilitated through opportunities to meet new people, the loosening of social taboos controlling sexual expression, a sense of anonymity that being away from home confers^5–8^ and in the context of engaging in risk behaviours such as alcohol and drug use, which may also change when away from home.^6^

Risk of sexually transmitted infection (STI)—in terms of both acquisition and onward transmission—depends on the characteristics of sexual partners and the STI prevalence in their places of residence. There is therefore the potential for those having sex while overseas to act as a bridge from areas of high to low STI/HIV prevalence. Information about the number of such partnerships and sexual mixing patterns is needed to inform modelling studies and to understand transmission dynamics more broadly.

Using data from the second National Survey of Sexual Attitudes & Lifestyles (Natsal-2), conducted in 1999–2001, we previously observed that 13.9% of men and 7.1% of women aged 16–44 years reported having sex while overseas in the past 5 years, and we examined associated factors and partners’ region of residence.^9^ This latest paper uses data from Natsal-3, conducted a decade later in 2010–2012, to update and extend these analyses to include people to age 74 years (reflecting the latest survey's broader conceptualisation of sexual health), and to explore the characteristics, behaviour and sexual health risks of those who report having new partners while overseas.

## Methods

### Participants and procedure

Natsal-3 is a stratified probability sample survey of 15 162 men and women aged 16–74 years, resident in Britain. The overall response rate was 57.7%. Interviews were carried out between September 2010 and August 2012. Participants were interviewed using computer-assisted personal interviewing with computer-assisted self-interview for the more sensitive questions, including those on sex while outside the UK. Sex was defined as vaginal, oral or anal with someone of the opposite sex, and oral, anal (for men) or genital contact (for women) with someone of the same sex. Further details of the methods have been published previously.^10^ ^11^ Data were collected on a range of socio-demographic variables and sexual and health behaviours.

Participants reporting one or more sexual partner(s) in the past 5 years were asked whether they had travelled outside the UK in the past 5 years and, if yes, were asked about partner(s) with whom they had had sex *for the first time* while outside the UK, including the number of such partners and their usual region of residence.

### Statistical analysis

Analyses were carried out using Stata (V.13) accounting for the stratification, clustering and weighting of the sample.^10^ ^11^ Weights were applied to account for unequal selection probabilities and non-response to correct for differences in sex, age group and region according to 2011 Census figures. Analyses were restricted to those reporting at least one sexual partner in the past 5 years (5259 men (weighted population prevalence of 85.9%) and 7271 women (weighted population prevalence of 82.1%)).

We calculated the percentage of the population who reported meeting new sexual partner(s) while overseas in the past 5 years as well as the mean and median numbers of these partners and the region of their home residence (so regardless of where sex occurred). We calculated the proportion of all partnerships in the past 5 years, which were newly formed while overseas by dividing the number of new partners while overseas by the total number of partners in the past 5 years (1) for the whole sample and (2) for those reporting new sexual partner(s) while overseas. CIs were bootstrapped as the resulting estimates were calculated from summary statistics and needed to have SEs estimated. Logistic regression models were used to explore the association between reporting new sexual partner(s) while overseas and a number of key socio-demographic and sexual and health behaviours. We present crude ORs and 95% CIs and ORs (and 95% CIs) adjusted for the socio-demographic characteristics found to be associated with reporting sex while overseas in the univariate analysis (ie, age, relationship status, ethnic category (White British vs other), social class and place of residence in Britain (Greater London vs other)) and number of partners in the past 5 years.

Participants who reported having sex while overseas were categorised according to the region of residence of the sexual partner(s), either UK-only partners or at least one non-UK partner. We also used logistic regression models to explore whether these two groups differed in terms of their socio-demographic characteristics and sexual and health behaviours, including after adjusting for age. Size of denominators precluded adjustment for more covariates.

Statistical significance was considered as p<0.05 for all analyses.

### Ethics

The Natsal-3 study was approved by the Oxfordshire Research Ethics Committee A (Ref: 10/H0604/27). Participants were provided with information about the survey and consented verbally to be interviewed.

## Results

### Frequency of reporting new sexual partner(s) in the past 5 years while overseas

Among those who had at least one sexual partner in the past 5 years, a higher proportion of men than women reported having sex with a new partner(s) while overseas during this period (9.2% of men and 5.3% of women; table 1). Among men and women who reported doing so, 35.0% and 24.9%, respectively, of their partnerships in the past 5 years had begun while they were overseas. In terms of partnerships (as opposed to individual participants), this corresponds to 9.3% and 4.6% of all partnerships reported by men and women with at least one partner in the past 5 years. The median number of partners overseas was one for men and women, with 95th percentile of 14 for men and 5 for women.

**Table 1:** Proportion reporting new sexual partner(s) while overseas in the past 5 years, and the number of new sexual partners while overseas, by gender and age group

Age group	16–74 year olds				16–34 year olds				35–74 year olds			
	Men		Women		Men		Women		Men		Women	
% (95% CI) reporting new sexual partners while overseas in the past 5 years												
1+ new partner while overseas	9.2%	(8.3 to 10.1)	5.3%	(4.8 to 5.8)	15.5%	(14.0 to 17.0)	10.7%	(9.6 to 11.8)	5.8%	(4.8 to 6.9)	2.2%	(1.8 to 2.8)
2+ new partners while overseas	4.5%	(3.9 to 5.2)	1.6%	(1.3 to 1.9)	8.0%	(6.9 to 9.3)	3.8%	(3.2 to 4.5)	2.6%	(2.0 to 3.5)	0.3%	(0.2 to 0.6)
5+ new partners while overseas	1.7%	(1.3 to 2.2)	0.3%	(0.2 to 0.4)	2.4%	(1.8 to 3.1)	0.6%	(0.4 to 0.9)	1.3%	(0.9 to 2.0)	0.1%	(0.0 to 0.4)
Denom. (unwt, wt)*	*5243, 6426*		*7257, 6275*		*2785, 2270*		*4072, 2260*		*2458, 4156*		*3185, 4015*	
Number of new sexual partners while overseas in the past 5 years												
Mean (SD)	3.4	(5.2)	2.0	(2.7)	2.9	(4.1)	2.0	(2.7)	4.2	(6.5)	1.7	(2.7)
Median (IQR)	1	1,3	1	1,2	2	1,3	1	1,2	1	1,4	1	1,1
5th, 95th percentile	1, 14		1, 5		1, 10		1, 5		1, 20		1, 4	
*Denom. (unwt, wt)*†	*588, 590*		*483, 331*		*439, 351*		*395, 241*		*149, 239*		*88, 90*	
% (95% CI) of all partnerships in the past 5 years who were new partners while overseas												
Of those with 1+ sexual partner in the past 5 years	9.3%	(9.2 to 9.5)	4.6%	(4.5 to 4.6)	8.6%	(8.5 to 8.8)	6.3%	(6.1 to 6.4)	10.2%	(9.8 to 10.6)	2.4%	(2.4 to 2.5)
Of those with 1+ sexual partner and new sex partner(s) while overseas in the past 5 years	35.0%	(34.3 to 35.8)	24.9%	(23.7 to 26.0)	27.1%	(26.7 to 27.6)	28.5%	(28.1 to 28.9)	51.3%	(49.0 to 53.7)	26.7%	(23.0 to 30.4)

*Denominator includes those who reported 1+ sexual partner in the past 5 years.

†Denominator includes those who reported 1+ sexual partner in the past 5 years and who reported new sex partner(s) while overseas in the past 5 years.

Younger (aged 16–34 years) men and women were more likely to report forming new partnerships while overseas (15.5% and 10.7% of younger men and women vs 5.8% and 2.2% of older men and women, aged at least 35 years, respectively). Of those with new partnerships made abroad, older men had a median of one partner in contrast to a median of two among younger men, but the distribution was more skewed for older men with 95th percentile of 20 vs 10, respectively. Overall, these partnerships constituted 51.3% of older men's partnerships in the past 5 years compared with 27.1% of younger men's partnerships. These differences were not seen for women.

### Factors associated with reporting new partner(s) while overseas

In univariate analyses, a number of socio-demographic factors including younger age, unmarried relationship status, not being of White British ethnicity, being a student and living in Greater London were identified as being associated with reporting forming new partner(s) while overseas (see online supplementary appendix A1 for men and online supplementary appendix A2 for women). In multivariable analyses (adjusting for socio-demographic characteristics as well as sexual partner numbers), only ethnic group remained associated for men but, for women, the associations with age, ethnic group, social class (less likely in those of lower socio-economic status) and living in Greater London remained.

10.1136/sextrans-2015-052459.supp1 Supplementary data

Reporting forming new partner(s) while overseas was also associated with a number of sexual behaviours including having sex with one or more new partner(s) without using a condom in the past year and, in the past 5 years, having larger numbers of partners (2 in 5 men with 10 or more partners reported new partner(s) while overseas), overlapping partnerships, same-sex partners (men), both same and opposite-sex partners (women) and with reporting new partner(s) while in the UK from outside the UK. Particularly strong associations were seen with paying for sex in the past 5 years and ever paying for sex outside the UK (both men only) with nearly three in five men who had paid for sex having new partner(s) while overseas (OR=17.45; 95% CI 12.47 to 24.42). There was a significant interaction with age with a stronger association observed for men aged 35 or over (OR=26.90; 95% CI 16.09 to 44.97 vs 11.69; 95% CI 7.54 to 18.10 for men aged under 35). After adjustment for demographic characteristics and partner numbers, associations remained for overlapping partnerships, and having new partner(s) in the UK from outside the UK (men and women), and with paying for sex in the past 5 years, and with ever paying for sex outside the UK (men only).

Associations were also seen with drug and alcohol use, including smoking, drinking alcohol over recommended limits and using illicit drugs in the past year. Associations with drinking and drug use persisted after adjustment. Men and women who reported attending a sexual health clinic, having had an HIV test or STI diagnosis/es, all within the past 5 years, were more likely to report having had new sexual partner(s) while overseas during this time. Associations were attenuated after adjustment and, although all remained associated in women, in men only an association with having an HIV test was seen. There was a strong association with both self-perceived HIV and STI risk but this was much reduced after adjustment.

### Sexual mixing patterns

Of the 1071 men and women who reported having new partner(s) while overseas, 71.9% (95% CI 67.6% to 75.9%) of men and 58.4% (95% CI 53.3% to 63.3%) of women reported that at least one partner was from outside the UK, while among men, 33.4% (95% CI 29.2% to 37.8%) and among women, 48.0% (95% CI 42.9% to 53.1%) reported that at least one new partner usually lived in the UK (table 2). A larger percentage of men than women reported having partners from other European countries (40.1% vs 28.5%) and from North America (11.8% vs 6.1%) and Asia (12.4% vs 3.9%), but men were less likely than women to report having partners from the Middle East/North Africa (2.4% vs 5.7%). Sexual mixing patterns varied by participant's ethnicity with participants most commonly reporting having partners from the geographical region concordant with their ethnicity (see online supplementary appendix A3).

**Table 2:** Country/region of residence of new sexual partner(s) while overseas in the past 5 years, by gender

Country/region	16–74 year olds				p Value	16–34 year olds				p Value	35–74 year olds				p Value
	Men		Women			Men		Women			Men		Women		
UK	33.4%	(29.2, 37.8)	48.0%	(42.9, 53.1)	<0.0001	39.5%	(34.5, 44.8)	45.7%	(40.1, 51.5)	0.1097	24.3%	(17.4, 32.8)	54.1%	(42.8, 64.9)	<0.0001
Other European country/ies	40.1%	(35.6, 44.8)	28.5%	(24.2, 33.3)	0.0003	41.9%	(37.0, 47.0)	30.5%	(25.7, 35.9)	0.0014	37.5%	(29.2, 46.5)	22.9%	(14.7, 33.9)	0.0341
Australia/New Zealand	7.8%	(5.6, 10.8)	6.5%	(4.4, 9.3)	0.4651	10.3%	(7.4, 14.1)	6.2%	(4.3, 9.0)	0.0456	4.1%	(1.5, 11.0)	7.2%	(2.9, 16.7)	0.4209
North America	11.8%	(9.1, 15.3)	6.1%	(4.0, 9.1)	0.007	13.0%	(9.9, 16.9)	6.1%	(3.9, 9.4)	0.0024	10.1%	(5.6, 17.8)	6.1%	(2.3, 15.2)	0.3963
South America	4.0%	(2.6, 6.2)	3.5%	(2.0, 6.3)	0.7246	5.5%	(3.4, 8.8)	4.8%	(2.7, 8.5)	0.7204	1.8%	(0.5, 6.0)	0.0%		0.3196
Caribbean	1.2%	(0.5, 2.9)	3.0%	(1.8, 5.1)	0.0654	1.4%	(0.5, 3.8)	3.3%	(1.9, 5.9)	0.1254	1.1%	(0.2, 4.7)	2.3%	(0.7, 6.9)	0.416
Asia	12.4%	(9.5, 15.9)	3.9%	(2.3, 6.7)	0.0001	9.0%	(6.3, 12.7)	4.8%	(2.7, 8.5)	0.0682	17.4%	(12.0, 24.5)	1.5%	(0.4, 6.2)	<0.0001
Middle East/North Africa	2.4%	(1.2, 4.6)	5.7%	(3.5, 9.0)	0.0357	1.7%	(0.7, 4.2)	3.8%	(1.8, 7.7)	0.1851	3.4%	(1.3, 8.6)	10.9%	(5.9, 19.2)	0.0294
African countries (other than North Africa)	4.5%	(2.9, 7.0)	2.9%	(1.7, 5.1)	0.2464	2.9%	(1.6, 5.0)	3.7%	(2.1, 6.5)	0.5386	6.9%	(3.6, 12.8)	0.9%	(0.1, 6.3)	0.0215
Other	0.7%	(0.2, 2.6)	1.8%	(0.9, 3.3)	0.198	0.4%	(0.1, 1.4)	1.7%	(0.9, 3.3)	0.0285	1.1%	(0.2, 7.5)	1.9%	(0.4, 8.1)	0.6578
*Denominator (unwt, wt)^1^*	*588, 590*		*483, 331*			*439, 351*		*395, 241*			*149, 239*		*88, 90*		

Percentages do not sum to 100% because respondents could report new partners from more than one country/region.

*Denominator includes those who reported 1+ sexual partner in the past 5 years and who reported new sexual partner(s) while overseas in the past 5 years.

### Profile of participants reporting new partner(s) while overseas by region of residence of new partner(s) (at least one partner not from the UK vs UK only)

Compared with those only reporting partners from the UK while overseas, those with at least one non-UK partner while overseas were more likely to be aged 25 or over (men) or 25–34 years (women), not in a steady relationship (women only), of White Other or non-White ethnicity (vs White British ethnicity), in managerial or professional occupations (vs intermediate or semiroutine/routine occupations) (men only) and live in Greater London (table 3 men and table 4 women). Associations persisted after adjustment for age. Among participants who had a partner who did not live in the UK, those who identified as ‘White Other’ or ‘non-White’ commonly (64.9%, 95% CI 56.6% to 72.4%) reported partner(s) who lived in a geographical region/country corresponding to the participant's ethnicity.

**Table 3:** Comparison of characteristics of men reporting forming new sexual partner(s) while overseas in the past 5 years by region of residence of partner (at least one non-UK vs UK only)

	Region of residence of partner								
	UK only		At least one non-UK						
Denominators	*174, 160*		*395, 410*						
	Per cent	95% CI	Per cent	95% CI	OR*	p Value	AOR*†	95% CI	p Value
*Socio-demographic variables*									
Age (years)						0.0427			
16–24	36.6	(29.1 to 44.9)	25.3	(21.0 to 30.0)	1				
25–34	29.6	(22.7 to 37.5)	32.1	(27.4 to 37.1)	1.57				
35+	33.8	(25.2 to 43.7)	42.7	(36.7 to 48.9)	1.83				
Relationship status						0.1081			0.1191
Married/civil partnership	25.0	(17.3 to 34.5)	26.5	(21.0 to 32.8)	1		1	–	
Living with partner	18.1	(12.1 to 26.2)	16.9	(13.1 to 21.6)	0.88		1.08	(0.51 to 2.28)	
In a ‘steady’ ongoing relationship but not living together	25.5	(19.3 to 32.9)	17.2	(13.5 to 21.7)	0.64		0.80	(0.41 to 1.56)	
Not in a ‘steady’ relationship	31.4	(24.3 to 39.6)	39.4	(34.1 to 45.0)	1.18		1.47	(0.80 to 2.71)	
Ethnicity						0.0004			0.0011
White British	85.9	(78.4 to 91.1)	65.0	(59.0 to 70.6)	1		1	–	
White other	4.0	(1.5 to 10.3)	14.6	(10.6 to 19.7)	4.86		5.02	(1.69 to 14.86)	
Non-White	10.1	(6.0 to 16.6)	20.4	(15.7 to 26.1)	2.66		2.57	(1.24 to 5.30)	
NSSEC code (individual socio-economic status)‡						0.0022			0.0026
Managerial and professional occupations	23.6	(17.1 to 31.6)	43.6	(38.0 to 49.4)	1		1	–	
Intermediate occupations	16.7	(11.1 to 24.2)	13.3	(9.9 to 17.6)	0.43		0.44	(0.23 to 0.87)	
Semiroutine/routine occupations	45.0	(36.6 to 53.8)	28.5	(23.5 to 34.1)	0.34		0.36	(0.21 to 0.61)	
No job (10+ h/week) or not in last 10 years	2.8	(1.1 to 6.5)	2.0	(0.9 to 4.5)	0.39		0.36	(0.10 to 1.23)	
Student in full-time education	12.0	(7.9 to 17.9)	12.6	(9.4 to 16.6)	0.56		0.67	(0.33 to 1.35)	
Resident in Greater London						0.0036			0.0023
No	88.9	(82.1 to 93.4)	75.5	(69.2 to 80.9)	1		1	–	
Yes	11.1	(6.6 to 17.9)	24.5	(19.1 to 30.8)	2.61		2.64	(1.42 to 4.92)	
*Sexual behaviours*									
No. of sexual partners, past 5 years						0.0018			0.0002
1	27.2	(19.4 to 36.8)	10.8	(7.7 to 15.1)	1		1	–	
2–4	24.1	(17.7 to 32.0)	26.7	(22.0 to 32.0)	2.78		3.34	(1.62 to 6.86)	
5–9	21.8	(16.2 to 28.7)	28.3	(23.4 to 33.8)	3.26		4.58	(2.25 to 9.32)	
10+	26.8	(20.2 to 34.7)	34.1	(28.8 to 39.8)	3.19		4.43	(2.23 to 8.77)	
Overlap between partners, past 5 years						0.0021			0.0014
No	61.6	(53.3 to 69.2)	45.4	(39.4 to 51.6)	1		1	–	
Yes	38.4	(30.8 to 46.7)	54.6	(48.4 to 60.6)	1.92		1.97	(1.30 to 2.97)	
1+ new unprotected partner, past year						0.7419			0.6976
No	68.1	(59.6 to 75.5)	66.5	(60.7 to 71.7)	1		1	–	
Yes	31.9	(24.5 to 40.4)	33.5	(28.3 to 39.3)	1.08		1.09	(0.70 to 1.70)	
Paid for sex, past 6 years						<0.0001			0.0001
No	90.8	(84.5 to 94.7)	70.0	(64.0 to 75.5)	1		1	–	
Yes	9.2	(5.3 to 15.5)	30.0	(24.5 to 36.0)	4.23		4.08	(2.06 to 8.11)	
Ever paid money for sex outside the UK						<0.0001			<0.0001
No	91.4	(85.8 to 95.0)	66.8	(60.8 to 72.3)	1		1	–	
Yes	8.6	(5.0 to 14.2)	33.2	(27.7 to 39.2)	5.31		5.17	(2.72 to 9.85)	
New partner in UK from outside UK, past 5 years						0.0003			0.0001
No	89.7	(83.9 to 93.6)	75.2	(70.1 to 79.8)	1		1	–	
Yes	10.3	(6.4 to 16.1)	24.8	(20.2 to 29.9)	2.88		3.05	(1.73 to 5.37)	
*Health behaviours*									
Smoker						0.9077			0.8423
No	66.2	(57.5 to 73.9)	65.6	(59.7 to 71.1)	1		1	–	
Yes	33.8	(26.1 to 42.5)	34.4	(28.9 to 40.3)	1.03		1.05	(0.67 to 1.62)	
Average alcoholic consumption, per week§						0.9354			0.9922
None	13.1	(8.1 to 20.6)	14.3	(10.3 to 19.5)	1		1	–	
Not more than recommended	75.3	(67.1 to 82.0)	73.5	(67.7 to 78.6)	0.9		0.96	(0.49 to 1.87)	
More than recommended	11.6	(7.3 to 17.9)	12.2	(8.8 to 16.7)	0.96		0.96	(0.40 to 2.26)	
Drug use, past year						0.3278			0.1363
No	68.7	(60.7 to 75.8)	65.5	(60.0 to 70.7)	1		1	–	
Yes, cannabis only	10.3	(6.5 to 15.8)	15.1	(11.5 to 19.4)	1.54		1.84	(1.01 to 3.36)	
Yes, drugs other than cannabis	21.0	(15.2 to 28.3)	19.4	(15.4 to 24.1)	0.97		1.12	(0.68 to 1.85)	
*Sexual health outcomes*									
Attended sexual health clinic, past 5 years						0.9281			0.8051
No	66.7	(58.3 to 74.1)	67.1	(61.4 to 72.4)	1		1	–	
Yes	33.3	(25.9 to 41.7)	32.9	(27.6 to 38.6)	0.98		1.05	(0.69 to 1.60)	
HIV test, past 5 years						0.0012			0.0004
Not in past 5 years/never	79.5	(72.0 to 85.4)	64.1	(58.3 to 69.6)	1		1	–	
In past 5 years	20.5	(14.6 to 28.0)	35.9	(30.4 to 41.7)	2.17		2.28	(1.45 to 3.60)	
STI diagnosis, past 5 years						0.1423			0.0885
No	90.3	(84.7 to 93.9)	85.6	(81.4 to 89.0)	1		1	–	
Yes	9.8	(6.1 to 15.3)	14.4	(11.0 to 18.6)	1.56		1.66	(0.93 to 2.98)	
*Risk perception*									
HIV/AIDS risk: to self						0.1641			0.1084
Greatly at risk/quite a lot	5.3	(2.6 to 10.3)	5.5	(3.6 to 8.2)	1.24		1.29	(0.55 to 3.03)	
Not very much	35.1	(27.6 to 43.5)	44.5	(39.0 to 50.3)	1.51		1.58	(1.03 to 2.42)	
Not at all at risk	59.6	(51.1 to 67.6)	50.0	(44.4 to 55.6)	1		1	–	
Other STI risk: to self						0.1876			0.0864
Greatly at risk/quite a lot	10.2	(6.5 to 15.5)	10.4	(7.4 to 14.5)	1.24		1.4	(0.77 to 2.57)	
Not very much	37.8	(30.0 to 46.2)	46.9	(40.9 to 52.9)	1.51		1.64	(1.05 to 2.56)	
Not at all at risk	52.1	(43.7 to 60.4)	42.7	(37.2 to 48.5)	1		1	–	

*ORs are for reporting at least one non-UK new partner while overseas versus reporting UK only new partner(s) while overseas.

†Adjusted for age.

‡NSSEC, National Statistics Socio-Economic Classification.^12^

§Recommended alcohol limits (21 units/week for men and 14 units/week for women) as defined by Royal College of Physicians.^13^

STI, sexually transmitted infection.

**Table 4:** Comparison of characteristics of women reporting forming new sexual partner(s) while overseas in the past 5 years by region of residence of partner (at least one non-UK vs UK only)

	Region of residence of partner								
	UK only		At least one non-UK						
Denominators	*197, 135*		*274, 189*						
	Per cent	95% CI	Per cent	95% CI	OR*	p Value	AOR*†	95% CI	p Value
Socio-demographic variables									
Age (years)						0.0039			
16–24	39.8	(32.2 to 47.8)	32.7	(26.7 to 39.3)	1				
25–34	28.1	(22.1 to 35.2)	44.4	(37.9 to 51.1)	1.92				
35+	32.1	(24.3 to 41.0)	22.9	(17.3 to 29.5)	0.87				
Relationship status						0.0002			0.0015
Married/civil partnership	32.5	(24.9 to 41.2)	16.0	(11.5 to 21.9)	1		1	–	
Living with partner	17.3	(12.1 to 24.1)	15.9	(11.2 to 22.1)	1.87		1.54	(0.71 to 3.31)	
In a ‘steady’ ongoing relationship but not living together	24.6	(18.9 to 31.4)	21.2	(16.0 to 27.4)	1.75		1.40	(0.70 to 2.83)	
Not in a ‘steady’ relationship	25.6	(19.4 to 32.9)	46.9	(39.9 to 54.1)	3.73		3.09	(1.64 to 5.80)	
Ethnicity						<0.0001			<0.0001
White British	82.3	(75.3 to 87.6)	61.9	(54.6 to 68.7)	1		1	–	
White other	3.9	(1.9 to 8.0)	18.6	(13.5 to 25.1)	6.34		5.94	(2.64 to 13.33)	
Non-White	13.8	(9.0 to 20.5)	19.4	(14.2 to 26.0)	1.87		1.73	(0.97 to 3.07)	
NSSEC code (individual socio-economic status)‡						0.1199			0.0687
Managerial and professional occupations	26.3	(20.0 to 33.8)	39.2	(32.2 to 46.7)	1		1	–	
Intermediate occupations	20.6	(14.6 to 28.4)	16.7	(11.9 to 22.9)	0.54		0.54	(0.29 to 1.02)	
Semiroutine/routine occupations	28.6	(22.0 to 36.2)	22.4	(17.1 to 28.8)	0.53		0.47	(0.27 to 0.83)	
No job (10+ h/week) or not in last 10 years	7.9	(4.3 to 14.0)	5.0	(2.8 to 9.0)	0.43		0.55	(0.23 to 1.31)	
Student in full-time education	16.6	(11.5 to 23.4)	16.6	(11.7 to 23.0)	0.67		0.47	(0.24 to 0.93)	
Resident in Greater London						0.0001			<0.0001
No	88.3	(81.5 to 92.8)	69.0	(61.9 to 75.3)	1		1	–	
Yes	11.7	(7.2 to 18.5)	31.0	(24.7 to 38.1)	3.38		3.35	(1.94 to 5.77)	
*Sexual behaviours*									
No. of sexual partners, past 5 years						0.0004			0.0070
1	37.5	(29.7 to 46.1)	16.3	(11.3 to 22.7)	1		1	–	
2–4	21.6	(16.0 to 28.4)	31.9	(26.0 to 38.4)	3.41		3.11	(1.61 to 6.02)	
5–9	23.0	(17.2 to 30.1)	31.6	(25.3 to 38.6)	3.17		2.78	(1.37 to 5.64)	
10+	17.9	(12.8 to 24.4)	20.3	(15.3 to 26.4)	2.62		2.27	(1.06 to 4.86)	
Overlap between partners, past 5 years						0.0125			0.0448
No	71.6	(64.7 to 77.6)	59.6	(52.7 to 66.2)	1		1	–	
Yes	28.4	(22.4 to 35.3)	40.4	(33.8 to 47.3)	1.71		1.54	(1.01 to 2.36)	
1+ new unprotected partner, past year						0.823			0.7958
No	71.1	(63.6 to 77.6)	70.1	(63.8 to 75.7)	1		1	–	
Yes	28.9	(22.4 to 36.4)	29.9	(24.3 to 36.2)	1.05		0.94	(0.60 to 1.47)	
New partner in UK from outside UK, past 5 years						0.0262			0.0159
No	89.6	(82.4 to 94.1)	79.3	(72.3 to 84.8)	1		1	–	
Yes	10.4	(5.9 to 17.6)	20.7	(15.2 to 27.7)	2.26		2.32	(1.17 to 4.60)	
*Health behaviours*									
Smoker						0.7252			0.8847
No	68.9	(61.6 to 75.5)	67.3	(60.9 to 73.2)	1		1	–	
Yes	31.1	(24.5 to 38.4)	32.7	(26.8 to 39.1)	1.08		0.97	(0.64 to 1.46)	
Average alcoholic consumption, per week§						0.2969			0.2138
** **None	18.9	(13.5 to 25.9)	25.8	(19.6 to 33.2)	1		1	–	
Not more than recommended	58.9	(51.0 to 66.4)	52.3	(45.5 to 59.0)	0.65		0.64	(0.38 to 1.07)	
More than recommended	22.2	(16.4 to 29.3)	21.9	(16.8 to 28.1)	0.72		0.63	(0.33 to 1.20)	
Drug use, past year						0.0014			0.0060
No	82.7	(76.0 to 87.8)	68.0	(61.2 to 74.2)	1		1	–	
Yes, cannabis only	6.8	(4.1 to 11.3)	18.2	(13.2 to 24.5)	3.23		2.92	(1.48 to 5.76)	
Yes, drugs other than cannabis	10.5	(6.5 to 16.5)	13.7	(9.7 to 19.1)	1.59		1.44	(0.77 to 2.70)	
*Sexual health outcomes*									
Attended sexual health clinic, past 5 years						0.0731			0.4111
No	63.2	(55.3 to 70.5)	53.8	(46.7 to 60.6)	1		1	–	
Yes	36.8	(29.5 to 44.7)	46.2	(39.4 to 53.3)	1.48		1.20	(0.78 to 1.84)	
HIV test, past 5 years						0.0104			0.0497
Not in past 5 years/never	68.0	(60.0 to 75.1)	54.4	(47.2 to 61.3)	1		1	–	
In past 5 years	32.0	(24.9 to 40.0)	45.6	(38.7 to 52.8)	1.78		1.55	(1.00 to 2.41)	
STI diagnosis, past 5 years						0.9939			0.4766
No	82.1	(75.2 to 87.4)	82.1	(75.9 to 87.0)	1		1	–	
Yes	17.9	(12.6 to 24.8)	17.9	(12.9 to 24.3)	1		0.82	(0.47 to 1.42)	
*Risk perception*									
HIV/AIDS risk: to self						0.5743			0.8726
Greatly at risk/quite a lot	4.7	(2.6 to 8.5)	4.4	(24 to 7.9)	1.01		0.92	(0.36 to 2.37)	
Not very much	33.9	(26.8 to 41.7)	39.1	(32.8 to 45.8)	1.25		1.11	(0.71 to 1.72)	
Not at all at risk	61.5	(53.6 to 68.8)	56.6	(49.7 to 63.2)	1		1	–	
Other STI risk: to self						0.0442			0.1660
Greatly at risk/quite a lot	6.4	(3.7 to 10.7)	9.7	(6.2 to 14.9)	1.94		1.69	(0.81 to 3.53)	
Not very much	32.8	(26.2 to 40.3)	42.5	(36.3 to 49.1)	1.65		1.46	(0.94 to 2.27)	
Not at all at risk	60.8	(52.9 to 68.1)	47.7	(41.1 to 54.5)	1		1	–	

*ORs are for reporting at least one non-UK new partner while overseas versus reporting UK only new partner(s) while overseas.

†Adjusted for age.

‡NSSEC, National Statistics Socio-Economic Classification.^12^

§Recommended alcohol limits (21 units/week for men and 14 units/week for women) as defined by Royal College of Physicians.^13^

STI, sexually transmitted infection.

In terms of sexual behaviours, those reporting at least one non-UK partner while overseas were more likely to report higher partner numbers, overlapping partnerships and new partner(s) in the UK from outside the UK, all in the past 5 years, but were not more likely to report unprotected sex with new partner(s) in the past year. There was a particularly strong association among men only between reporting non-UK partner(s) and paying for sex in the past 5 years (OR=4.23; 95% CI 2.20 to 8.15)) and ever having paid for sex outside the UK (OR=5.31; 95% CI 2.87 to 9.82). Of those men reporting non-UK partner(s), 26.7% (95% CI 21.6 to 32.5) had both paid for sex in the past  years and had ever paid for sex abroad. This proportion was higher in older (35–74 years) than younger men (16–34 years): 36.9% (95% CI 27.3% to 47.7%) vs 19.1% (95% CI 14.5% to 24.7%). These men reported a median of three partners while overseas (95th percentile: 21) in contrast to a median of one (95th percentile: 11) for those not reporting both paying for sex in the past 5 years and ever having paid for sex abroad.

We found few associations with health and health-seeking behaviours. Men reporting non-UK partner(s) were more likely to have had an HIV test in the past 5 years and this association persisted after adjustment for age (AOR 2.28) and after additional adjustment for number of partners and ethnicity (AOR=1.93; 95% CI 1.19 to 3.14)). In unadjusted analysis, there was an association among women with using cannabis in the past year, having had an HIV test in the past 5 years and self-perceived STI risk. Associations with drug use and HIV testing persisted after adjustment for age.

## Discussion

### Principal findings

Around 1 in 10 men and 1 in 20 women in Britain reported forming a new sexual partnership while overseas in the past 5 years. Men and women who had new partner(s) while overseas were more likely to report a range of sexual risk behaviours as well as STI diagnosis/es, but for men, this was no longer the case after adjusting for the number of sexual partners reported, demonstrating the mechanism through which this increased. For the majority of those who had new partnership(s) abroad, at least one of these partners lived outside the UK. This group differed in terms of some key characteristics and behaviours compared with those whose partners were from the UK only. They were less likely to identify as ‘White (British)’ and reported more partners, paid partners (men only), partners from outside the UK while in the UK and having tested for HIV. The men tended to be older (over 35 years) but women were more likely to be aged 25–34 years.

### Strengths and weaknesses of the study

Given the broad remit of Natsal, the number of questions asked about participants’ experience of sex while overseas was limited. Of note, we do not have information on number and types of sex acts, whether condoms were used with these partners, and we are unable to tell whether STIs reported in the same time period were acquired while overseas. We were able to compare the socio-demographic characteristics and behaviours of those who had partner(s) living outside the UK with those whose partners while overseas were from the UK only. However, the limited numbers reporting non-UK partners precluded further analyses, and we were unable to explore reasons why individuals form new partnerships abroad, which might include sex tourism, having sex with other UK residents while on holiday abroad and, among migrants to the UK, having sex with partners in their country of origin. We could only approximate the migrant population as those reporting partners from a geographical region that corresponded to their ethnicity. Furthermore, we recognise that some people had sex overseas *before* they moved to the UK. Additionally, we only know the regions where participants reported their partners were from, and not the number of new partnerships formed with people from each region. Finally, although we asked about paying for sex overseas, we did not restrict this to the past 5 years, nor did we ask about other forms of transactional sex.

### Strengths and weaknesses with respect to other studies and important differences in results

In a recent systematic review, the prevalence of foreign travel-associated casual sex from all studies was estimated to be about 20% (of participants), and condoms were not used in around half of these encounters.^14^ However, this review synthesised studies reporting on sex which occurred during varying time periods and was largely based on convenience samples of returning travellers or clinic studies, many of which were of low quality.

Despite the increase in international travel,^1^ the proportion of men and women aged 16–44 years reporting new partnership(s) while overseas in Natsal-3 was similar to that observed a decade earlier in Natsal-2 (12.5% (95% CI 11.3% to 13.9%) vs 13.9% for men and 7.6% (985% CI 6.8% to 8.4%) vs 7.1% for women, respectively), as was the number of new partners reported and the proportion of men and women (aged 16–44 years) reporting having at least one non-UK partner while overseas (8.6% vs 9.6% for men and 4.6% vs 4.7% for women).^9^ We also found similar associations with socio-demographic and behavioural measures and reporting new partner(s) while overseas as were observed in Natsal-2.

### Meaning of the study, possible explanations and implications for clinicians and policymakers

This paper is important for informing STI transmission models since it demonstrates the extent to which sexual partnerships are not formed among a ‘closed’ population. However, these data suggest that the prevalence of reporting new partner(s) while overseas has not changed over the past 10 years. At a population level, those reporting new partners while overseas continue to be a high-risk group, being more likely to report sexual risk behaviours as well as potentially harmful health behaviours, including drug and alcohol use. Sex while overseas may itself be less likely to be protected with the ‘freedom’ of travel, and also potentially in the context of other behaviours like alcohol and drug use, which may prevent people from adopting safer-sex behaviours,^6^ putting them at risk of transmitting and acquiring STIs,^15^ ^16^ as well as other adverse sexual health outcomes including unintended pregnancy and sexual violence.^17^ This argues for the importance of holistic travel advice addressing sex in the context of broader health behaviours.

People clearly have sex while overseas in a variety of contexts, and for some this will not put them at higher risk of STIs or other negative health consequences but instead simply reflects trends in international migration and ethnic mixing. In this context, individuals having sex with new partners while overseas take on the STI/HIV risk of the country their partner is living in and thus may potentially act as a bridge between areas of high and low STI/HIV prevalence.^2^ ^3^ ^18^ As such, for some migrant populations sex while overseas may be an important risk factor for STI/HIV transmission in its own right, aside from other sexual risk behaviours reported. Other studies report a high prevalence of unprotected sex in migrants travelling home^18^ arguing for the provision of culturally appropriate STI/HIV prevention messages for migrant populations.

Young people were more likely to report forming partnerships while overseas and appropriate health promotion information should be available for this age group. However, 1 in 20 men and 1 in 40 women aged 35 and over reported new partner(s) while overseas in the past 5 years, and a group of older men reported high numbers of partners while abroad. These proportions are likely to increase as older people maintain good health, have the financial means to travel and are now more likely to experience partnership breakdown,^19^ and so older age groups should also be considered for health promotion messages by health professionals when consulting for travel advice.

While we found that the geographical regions in which participants’ partners lived largely reflected travel trends, some regions, for example, Asia, featured more frequently than would be expected, suggesting sex tourism. Around a quarter of men reporting partner(s) not from the UK had paid for sex in the past 5 years *and* had ever paid for sex abroad versus around 6% of men only reporting partners from the UK while overseas. This proportion was higher in older men. Men who pay for sex are an important core group for STI transmission, not necessarily via their paid partners but through other high-risk behaviours.^20^

### Unanswered questions and future research

Given demographic changes, it is important that we monitor trends in sex while overseas across the life course as well as over time. Because of its association with higher risk sexual behaviour, research is also needed to examine the context of having sex while overseas to establish whether—and if so, how—travel abroad affects sexual behaviour. Work is also needed to establish the best ways of communicating safe sex messages in pretravel advice since previous interventions have shown limited effectiveness.^21^

## Conclusion

Those reporting new partner(s) while overseas were at higher sexual risk overall, but those reporting having sex abroad are a heterogeneous group so travel advice should include, as standard, sexual health as part of holistic health advice for all travellers, regardless of age, destination or motivation for travel.

**Key messages**

- Men and women reporting new partner(s) while overseas were more likely to report a range of harmful health behaviours, including sexual risk and substance use.
- For the majority of those who formed new partnership(s) abroad at least one of these partners lived outside the UK.
- With international travel on the increase, and people travelling for many reasons, sexual health advice should be included as part of holistic health advice for all travellers.
