# Supplementary material for: Forming new sex partnerships while overseas: findings from the third British National Survey of Sexual Attitudes & Lifestyles (Natsal-3)
Source: Sex Transm Infect. 2016 Jun 6;92(6):415–23. doi: 10.1136/sextrans-2015-052459 (PMC5013104; doi:10.1136/sextrans-2015-052459)
Supplement: Supplementary data [file sextrans-2015-052459supp.pdf]

Web Appendix 1: Variations in the % of men reporting new sexual partner(s) while overseas in the past five years by key demographics and sexual and health behaviours

	%	(95%CI)	Crude OR	(95%CI)	p-value	AOR [1]	(95%CI)	p-value	Denom. (unwt, wt) [2]
**All men**	9.2%	(8.3-10.1)							*5243, 6426*
Socio-demographic variables									
**Age (years)**					<0.0001			0.321	
16-24	16.9%	(14.8-19.2)	1	-		1	-		*1354, 988*
25-34	14.4%	(12.6-16.5)	0.83	(0.67-1.03)		1.14	(0.86-1.50)		*1431, 1282*
35-44	7.6%	(5.8-10.0)	0.41	(0.29-0.56)		1.06	(0.72-1.56)		*761, 1354*
45-54	6.1%	(4.4-8.4)	0.32	(0.22-0.47)		0.94	(0.60-1.48)		*696, 1282*
55-64	3.5%	(2.2-5.4)	0.18	(0.11-0.29)		0.64	(0.36-1.11)		*591, 953*
65-74	4.2%	(2.5-6.9)	0.22	(0.13-0.38)		1.19	(0.62-2.30)		*410, 567*
**Relationship status**					<0.0001			0.5308	
Married/civil partnership	4.6%	(3.7-5.7)	1	-		1	-		*1960, 3432*
Living with partner	9.7%	(7.8-12.1)	2.26	(1.62-3.15)		0.97	(0.64-1.47)		*857, 1028*
In a 'steady' ongoing relationship but not living together	14.5%	(12.1-17.4)	3.56	(2.62-4.86)		0.97	(0.64-1.46)		*956, 765*
Not in a 'steady' relationship	18.6%	(16.3-21.1)	4.79	(3.64-6.31)		1.17	(0.80-1.72)		*1454, 1186*
**Ethnicity**					<0.0001			<0.0001	
White British	7.8%	(7.0-8.6)	1	-		1	-		*4430, 5354*
White Other	20.3%	(15.1-26.8)	3.02	(2.07-4.40)		2.38	(1.49-3.80)		*268, 335*
Mixed	13.8%	(7.8-23.2)	1.9	(1.00-3.60)		1.1	(0.58-2.09)		*100, 104*
Asian/Asian British	11.6%	(7.9-16.6)	1.55	(1.01-2.38)		1.99	(1.22-3.25)		*226, 352*
Black/Black British	16.4%	(10.8-24.2)	2.33	(1.42-3.83)		1.25	(0.70-2.23)		*154, 200*
Chinese/Other	26.9%	(15.4-42.6)	4.36	(2.18-8.73)		4.57	(1.97-10.60)		*56, 67*
**NSSEC code (individual socio-economic status)[3]**					<0.0001			0.1928	
Managerial & prof occupations	9.1%	(7.7-10.7)	1	-		1	-		*1731, 2445*
Intermediate occupations	7.9%	(6.2-9.9)	0.85	(0.63-1.16)		0.77	(0.55-1.09)		*824, 1100*
Semi-routine/routine occupations	9.4%	(8.0-10.9)	1.03	(0.81-1.32)		0.8	(0.60-1.05)		*1803, 2077*
No job(10+ hrs/week) or not in last 10	4.0%	(2.2-7.3)	0.42	(0.22-0.80)		0.47	(0.22-0.99)		*286, 316*

years									
Student in full-time education	15.7%	(12.5-19.6)	1.86	(1.37-2.53)		0.79	(0.54-1.17)		*582, 468*
**Resident in Greater London**					0.0001			0.168	
No	8.4%	(7.6-9.3)	1	-		1	-		*4717, 5590*
Yes	14.3%	(11.2-18.2)	1.82	(1.34-2.48)		1.28	(0.90-1.82)		*526, 836*
Sexual behaviours									
**No. of sexual partners, past five years**					<0.0001			<0.0001	
1	2.3%	(1.8-3.0)	1	-		1	-		*2632, 3931*
2-4	10.3%	(8.6-12.2)	4.77	(3.42-6.64)		4.45	(3.01-6.56)		*1435, 1457*
5-9	27.6%	(23.7-31.9)	15.89	(11.43-22.09)		14.08	(9.32-21.28)		*646, 561*
10+	42.2%	(37.1-47.4)	30.39	(21.55-42.86)		26.64	(17.44-40.70)		*512, 456*
**Overlap between partners, past five years**					<0.0001			0.0176	
No	5.6%	(4.9-6.4)	1	-		1	-		*4176, 5345*
Yes	27.4%	(24.2-30.9)	6.38	(5.13-7.93)		1.37	(1.06-1.78)		*1058, 1066*
**Same sex partners, past five years**					<0.0001			0.1158	
No	8.8%	(7.9-9.7)	1	-		1	-		*5054, 6236*
Yes, only same sex	27.8%	(18.3-39.9)	4.01	(2.34-6.88)		1.46	(0.81-2.63)		*106, 103*
Yes, and opposite sex	18.6%	(10.5-30.9)	2.38	(1.22-4.64)		0.61	(0.32-1.17)		*76, 79*
**1+ new unprotected partner, past year**					<0.0001			0.2509	
No	7.2%	(6.4-8.1)	1	-		1	-		*4233, 5452*
Yes	20.9%	(18.0-24.2)	3.41	(2.73-4.27)		1.17	(0.90-1.52)		*963, 918*
**Paid for sex, past five years**					<0.0001			<0.0001	
No	7.2%	(6.5-8.0)	1	-		1	-		*5015, 6176*
Yes	57.6%	(49.6-65.2)	17.45	(12.47-24.42)		6.71	(4.36-10.34)		*227, 249*
**Ever paid money for sex outside the UK**					<0.0001			<0.0001	
No	7.3%	(6.5-8.1)	1	-		1	-		*4884, 5961*
Yes	33.4%	(28.0-39.4)	6.39	(4.84-8.43)		5.46	(3.92-7.59)		*359, 465*
**New partner in UK from outside UK, past five years**					<0.0001			<0.0001	
No	7.6%	(6.8-8.5)	1	-		1	-		*4951, 6153*
Yes	44.5%	(37.8-51.5)	9.74	(7.17-13.21)		2.64	(1.88-3.71)		*291, 272*
Health behaviours									
**Smoker**					0.001			0.2202	

No	8.3%	(7.4-9.3)	1	-		1	-		*3629, 4678*
Yes	11.5%	(9.8-13.5)	1.44	(1.16-1.79)		0.85	(0.66-1.10)		*1614, 1747*
**Average alcoholic consumption, per week[4]**					0.0037			0.0473	
None	6.7%	(5.1-8.8)	1	-		1	-		*961, 1216*
Not more than recommended	9.4%	(8.5-10.5)	1.45	(1.06-1.98)		1.29	(0.90-1.84)		*3785, 4613*
More than recommended	12.6%	(9.8-16.1)	2	(1.33-3.00)		1.81	(1.12-2.91)		*474, 573*
**Drug use, past year**					<0.0001			0.0118	
No	7.3%	(6.5-8.2)	1	-		1	-		*4107, 5358*
Yes, cannabis only	13.9%	(11.1-17.2)	2.05	(1.54-2.73)		1.01	(0.73-1.39)		*618, 583*
Yes, drugs other than cannabis	24.4%	(20.3-28.9)	4.08	(3.16-5.28)		1.52	(1.15-2.03)		*504, 466*
Sexual health outcomes									
**Attended sexual health clinic, past five years**					<0.0001			0.1747	
No	6.9%	(6.1-7.8)	1	-		1	-		*4255, 5563*
Yes	24.2%	(20.9-27.7)	4.28	(3.41-5.37)		1.21	(0.92-1.59)		*909, 778*
**HIV test, past five years**					<0.0001			0.0005	
Not in past five years/never	7.2%	(6.4-8.1)	1	-		1	-		*4276, 5392*
In past five years	23.8%	(20.4-27.5)	4.02	(3.19-5.07)		1.59	(1.23-2.06)		*719, 744*
**STI diagnosis, past five years**					<0.0001			0.241	
No	8.3%	(7.4-9.2)	1	-		1	-		*4905, 6095*
Yes	28.4%	(22.7-35.0)	4.41	(3.19-6.09)		1.24	(0.86-1.79)		*278, 256*
Risk perception									
**HIV/AIDS risk: to self**					<0.0001			0.1993	
Greatly at risk / quite a lot	15.6%	(11.1-21.5)	2.66	(1.76-4.03)		0.85	(0.51-1.41)		*215, 206*
Not very much	17.4%	(15.1-20.0)	3.05	(2.47-3.77)		1.2	(0.94-1.53)		*1372, 1400*
Not at all at risk	6.5%	(5.7-7.4)	1	-		1	-		*3628, 4788*
**Other STI risk: to self**					<0.0001			0.0879	
Greatly at risk / quite a lot	24.7%	(19.1-31.1)	5.38	(3.78-7.65)		1.13	(0.75-1.70)		*281, 243*
Not very much	17.2%	(15.0-19.6)	3.41	(2.75-4.24)		1.33	(1.03-1.72)		*1496, 1522*
Not at all at risk	5.7%	(5.0-6.6)	1	-		1	-		*3440, 4630*

[1] Adjusted ORs adjusted for age (continuous), relationship status, ethnicity (White British vs. Other), social class, place of residence in Britain & partners in the past five years
[2] Denominators are for crude ORs
[3] NSSEC = National Statistics Socio-Economic Classification.[12]
[4] Recommended alcohol limits (21 units/week for men and 14 units/week for women) -as defined by Royal College of Physicians [13]

Web Appendix 2: Variations in the % of women reporting new sexual partner(s) while overseas in the past five years by key demographics and sexual and health behaviours

	%	(95%CI)	Crude OR	(95%CI)	p-value	AOR [1]	(95%CI)	p-value	Denom. (unwt, wt) [2]
**All women**	5.3%	(4.8-5.8)							*7257, 6275*
Socio-demographics									
**Age (years)**					<0.0001			0.0001	
16-24	12.3%	(10.6-14.3)	1	-		1	-		*1719, 959*
25-34	9.4%	(8.2-10.8)	0.74	(0.59-0.93)		1.31	(0.96-1.78)		*2353, 1301*
35-44	2.5%	(1.8-3.5)	0.18	(0.12-0.27)		0.51	(0.31-0.84)		*1139, 1378*
45-54	2.2%	(1.5-3.4)	0.16	(0.10-0.26)		0.61	(0.37-1.00)		*965, 1278*
55-64	1.8%	(1.1-3.0)	0.13	(0.08-0.23)		0.71	(0.38-1.33)		*694, 891*
65-74	2.3%	(1.1-4.6)	0.17	(0.08-0.35)		1.12	(0.48-2.57)		*387, 468*
**Relationship status**					<0.0001			0.1163	
Married/civil partnership	2.3%	(1.8-2.9)	1	-		1	-		*2843, 3370*
Living with partner	5.2%	(4.1-6.7)	2.37	(1.67-3.36)		1.03	(0.71-1.50)		*1294, 1023*
In a 'steady' ongoing relationship but not living together	9.3%	(7.7-11.2)	4.4	(3.21-6.01)		1.12	(0.77-1.62)		*1369, 794*
Not in a 'steady' relationship	11.7%	(10.0-13.6)	5.67	(4.24-7.57)		1.42	(1.00-2.00)		*1736, 1081*
**Ethnicity**					<0.0001			<0.0001	
White British	4.4%	(3.9-5.0)	1	-		1	-		*6082, 5247*
White Other	11.7%	(8.6-15.8)	2.85	(1.97-4.14)		2.65	(1.75-4.02)		*390, 346*
Mixed	8.5%	(4.5-15.8)	2.01	(1.00-4.04)		0.88	(0.40-1.92)		*170, 120*
Asian/Asian British	10.3%	(7.3-14.5)	2.48	(1.63-3.77)		3.91	(2.40-6.37)		*300, 279*
Black/Black British	5.4%	(2.8-10.1)	1.22	(0.61-2.42)		0.82	(0.38-1.77)		*217, 201*
Chinese/Other	10.3%	(5.1-19.8)	2.48	(1.16-5.31)		3.17	(1.49-6.71)		*84, 69*
**NSSEC code (individual socio-economic status)[3]**					<0.0001			0.0297	
Managerial & prof occupations	5.4%	(4.5-6.4)	1	-		1	-		*2174, 2070*
Intermediate occupations	4.6%	(3.6-5.9)	0.84	(0.61-1.16)		0.77	(0.55-1.09)		*1460, 1307*
Semi-routine/routine occupations	4.8%	(4.0-5.9)	0.89	(0.67-1.19)		0.68	(0.50-0.93)		*2084, 1685*

No job(10+ hrs/week) or not in last 10 years	2.7%	(1.8-4.2)	0.49	(0.30-0.80)		0.48	(0.28-0.83)		*771, 732*
Student in full-time education	12.2%	(9.4-15.6)	2.44	(1.71-3.48)		0.83	(0.56-1.25)		*729, 446*
**Resident in Greater London**					<0.0001			0.0309	
No	4.7%	(4.2-5.2)	1	-		1	-		*6488, 5479*
Yes	9.5%	(7.6-11.8)	2.16	(1.65-2.82)		1.43	(1.03-1.97)		*769, 796*
Sexual behaviours									
**No. of sexual partners, past five years**					<0.0001			<0.0001	
1	1.9%	(1.5-2.4)	1	-		1	-		*4342, 4427*
2-4	7.2%	(6.0-8.6)	3.98	(2.93-5.41)		3.19	(2.24-4.56)		*1893, 1249*
5-9	23.3%	(19.7-27.5)	15.58	(11.35-21.38)		11.32	(7.51-17.07)		*658, 391*
10+	33.6%	(27.9-39.9)	25.93	(18.22-36.90)		21.33	(13.95-32.60)		*338, 186*
**Overlap between partners, past five years**					<0.0001			0.0033	
No	3.7%	(3.3-4.2)	1	-		1	-		*6337, 5692*
Yes	20.6%	(17.8-23.7)	6.66	(5.34-8.29)		1.48	(1.14-1.91)		*899, 564*
**Same sex partners, past five years**					<0.0001			0.5299	
No	5.0%	(4.5-5.5)	1	-		1	-		*6906, 6033*
Yes, only same sex	2.2%	(0.7-7.1)	0.43	(0.13-1.46)		0.49	(0.14-1.75)		*79, 66*
Yes, and opposite sex	17.3%	(12.5-23.5)	4.01	(2.69-5.97)		0.93	(0.61-1.44)		*263, 170*
**1+ new unprotected partner, past year**					<0.0001			0.5389	
No	4.3%	(3.8-4.8)	1	-		1	-		*5996, 5455*
Yes	12.6%	(10.6-14.9)	3.23	(2.56-4.08)		0.91	(0.69-1.22)		*1214, 775*
**New partner in UK from outside UK, past five years**					<0.0001			<0.0001	
No	4.5%	(4.0-5.0)	1	-		1	-		*7025, 6106*
Yes	34.1%	(27.1-41.8)	11.05	(7.83-15.60)		4.3	(2.69-6.88)		*227, 165*
Health behaviours									
**Smoker**					0.0003			0.2827	
No	4.7%	(4.2-5.4)	1	-		1	-		*5166, 4744*
Yes	6.9%	(5.9-8.1)	1.49	(1.20-1.84)		0.88	(0.69-1.11)		*2091, 1531*
**Average alcoholic consumption, per week[4]**					<0.0001			0.0252	
None	4.1%	(3.3-5.1)	1	-		1	-		*2182, 1896*

Not more than recommended	5.0%	(4.3-5.7)	1.21	(0.92-1.59)		1.09	(0.81-1.46)		*4161, 3624*
More than recommended	9.8%	(8.0-12.0)	2.53	(1.86-3.45)		1.49	(1.09-2.04)		*885, 733*
**Drug use, past year**					<0.0001			0.0245	
No	4.2%	(3.8-4.8)	1	-		1	-		*6475, 5775*
Yes, cannabis only	15.6%	(11.8-20.3)	4.18	(2.95-5.91)		1.52	(1.02-2.25)		*431, 281*
Yes, drugs other than cannabis	19.9%	(15.3-25.3)	5.61	(3.97-7.93)		1.64	(1.08-2.47)		*337, 207*
Sexual health outcomes									
**Attended sexual health clinic, past five years**					<0.0001			0.0059	
No	3.5%	(3.0-3.9)	1	-		1	-		*5793, 5393*
Yes	16.4%	(14.2-18.9)	5.5	(4.39-6.88)		1.48	(1.12-1.96)		*1395, 832*
**HIV test, past five years**					<0.0001			0.001	
Not in past five year/never	3.9%	(3.4-4.4)	1	-		1	-		*5264, 4891*
In past five years	12.6%	(10.8-14.6)	3.55	(2.83-4.46)		1.53	(1.19-1.98)		*1547, 1010*
**STI diagnosis, past five years**					<0.0001			0.001	
No	4.5%	(4.0-5.0)	1	-		1	-		*6797, 5994*
Yes	24.9%	(19.9-30.7)	7.09	(5.18-9.71)		1.89	(1.29-2.75)		*409, 237*
**Non-volitional sex, aged 13+, past five years**					<0.0001			0.6649	
No	5.0%	(4.5-5.6)	1	-		1	-		*6917, 6057*
Yes	19.3%	(12.6-28.4)	4.53	(2.70-7.61)		1.14	(0.64-2.04)		*155, 82*
Risk perception									
**HIV/AIDS risk: to self**					<0.0001			0.0551	
Greatly at risk / quite a lot	9.4%	(6.0-14.3)	2.59	(1.59-4.22)		0.9	(0.52-1.58)		*203, 162*
Not very much	12.0%	(10.3-14.0)	3.42	(2.75-4.25)		1.34	(1.04-1.72)		*1388, 1002*
Not at all at risk	3.8%	(3.4-4.4)	1	-		1	-		*5630, 5082*
**Other STI risk: to self**					<0.0001			0.0904	
Greatly at risk / quite a lot	16.0%	(11.3-22.2)	5.16	(3.40-7.84)		1.11	(0.70-1.76)		*243, 169*
Not very much	11.2%	(9.7-12.9)	3.4	(2.75-4.19)		1.31	(1.03-1.67)		*1579, 1116*
Not at all at risk	3.6%	(3.1-4.1)	1	-		1	-		*5400, 4960*

[1] Adjusted ORs adjusted for age (continuous), relationship status, ethnicity (White British vs. Other), social class, place of residence in Britain & partners in the past five years
[2] Denominators are for crude ORs
[3] NSSEC = National Statistics Socio-Economic Classification.[12]
[4] Recommended alcohol limits (21 units/week for men and 14 units/week for women) as defined by Royal College of Physicians [13]

Web Appendix 3: Country/region of residence of new sexual partner(s) while overseas in the past 5 years, by participant's ethnicity

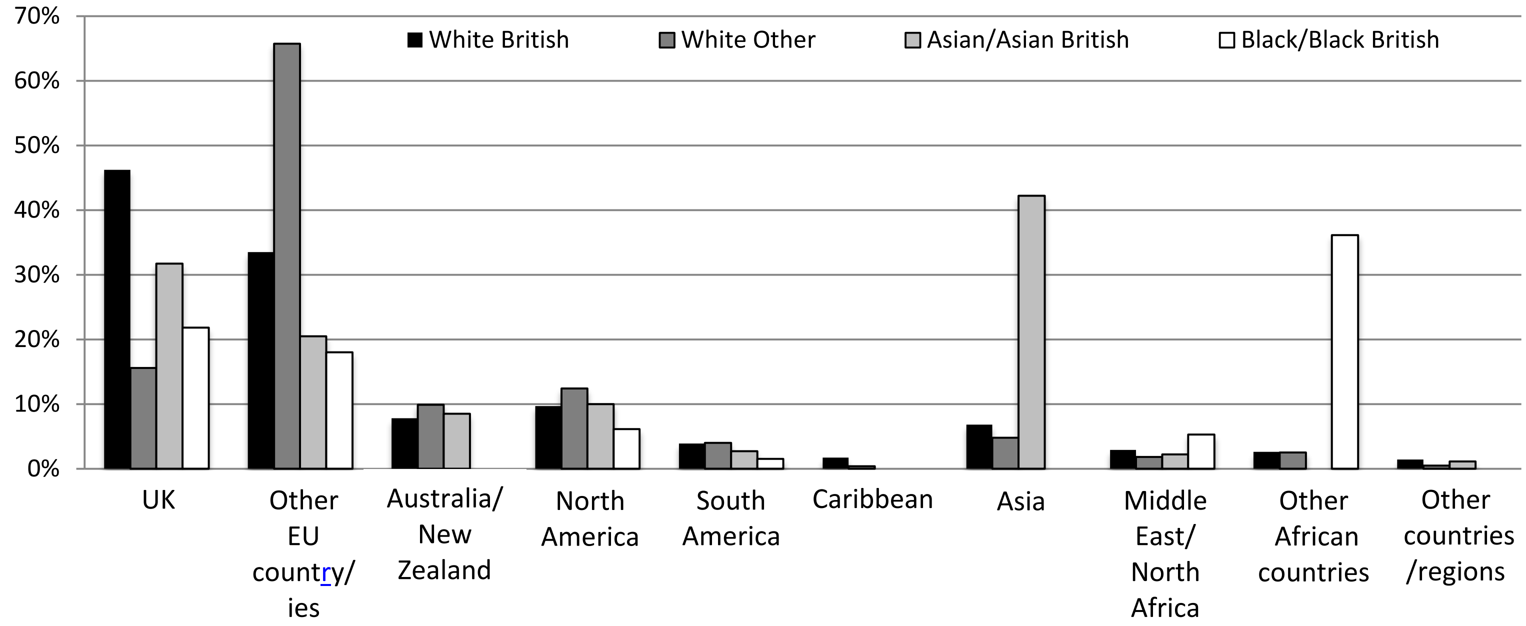

Percentages do not sum to 100% because respondents could report new partners from more than one country/region
Denominators too small for those self-identifying as 'Mixed' ethnicity or 'Chinese/Other' ethnicity
